# Beyond stereotypes of cerebral palsy: Exploring the lived experiences of young Canadians

**DOI:** 10.1111/cch.12705

**Published:** 2019-07-24

**Authors:** Julia E. Hanes, Oksana Hlyva, Peter Rosenbaum, Matthew Freeman, Tram Nguyen, Robert J. Palisano, Jan Willem Gorter

**Affiliations:** ^1^ CanChild Centre for Childhood Disability Research, Department of Pediatrics McMaster University Hamilton Ontario Canada; ^2^ School of Rehabilitation Science, CanChild Centre for Childhood Disability Research McMaster University Hamilton Ontario Canada; ^3^ Department of Physical Therapy and Rehabilitation Sciences Drexel University Philadelphia PA USA

**Keywords:** adult, cerebral palsy, healthcare experience, ICF, identity, lifecourse, patient‐oriented research, patient reviewer, patients, paediatrics, rehabilitation medicine

## Abstract

**Background:**

Health for people with cerebral palsy (CP) must extend beyond physical impairments to include social, environmental, and psychological factors that are rarely captured by quantitative research alone. This qualitative study sought to explore the lived experience of young people with CP with their physical, mental, and emotional health in the context of a larger longitudinal Canadian study focusing on brain function, physical and mental health, and well‐being.

**Methods:**

An integrated research team (including people with CP or other impairments, clinicians, and researchers) was formed to study participant‐identified research needs. A purposive sample of 16 people with CP (seven female), aged 17–29, Gross Motor Function Classification System (GMFCS) levels I–V, participated in three focus groups that were conceptualized and analysed using interpretive description methodology.

**Results:**

This study reports the experiences of people with CP across GMFCS levels and identifies some consequences of growing up with the condition: physical and mental health issues, importance of meaningful participation, impact of the environment, and identity formation. Participants shared challenges related to accessibility, healthcare, social/environmental supports, relationships, and sustainable employment.

**Discussion:**

Body structure and function challenges impact participation in activities of daily living, threatening participants' ability to form positive identities and live meaningful lives. People with CP desire to work but may require additional training, accommodation, and support to do so. Environmental conditions, including relationships, supportive people, and accessibility, shape participants' health, well‐being, and social/civic engagement. This study confirms the need for improved care for adults with CP, including multidisciplinary adult health team(s) and community services.

**Key Messages**

- Including lived experiences in research enriches our understanding of health and development.
- Using a lifecourse health development approach is essential for individuals with CP and should inform child and adult care services.
- Comprehensive care is essential for young people (adolescents/adults) given their medical needs and the multifaceted aspects of well‐being.
- Medical care and counselling people with CP needs to include discussions about mental health, pain, and sexual health/identity, especially in young adulthood.

## 1 INTRODUCTION

Health—formally defined by Huber et al. as “the ability to adapt and self‐manage”—is a multidimensional concept integrating physical, emotional, social, and environmental aspects of people's lives (Huber et al., 2011; Kim & Fox, 2006; World Health Organization, 2002). For people with childhood‐onset disabilities such as cerebral palsy (CP), health challenges continue and evolve in adulthood. CP refers to a group of nonprogressive conditions that impact movement and posture and are associated with activity limitations (Rosenbaum et al., 2007). Despite being the most common childhood physical disability, there is a paucity of research about the long‐term health outcomes of people with CP (Rosenbaum et al., 2007). This is especially true for aspects of health beyond the primary stereotypical motor impairments in children with CP and includes physical and mental health issues in adults.

It is essential to understanding the health and well‐being of people with CP as they age to ensure they are on a healthy trajectory over the lifecourse (Gorter et al., 2014; Palisano et al., 2017). Emerging quantitative literature describes some aspects of the health outcomes of adults with CP, including life expectancy (Day, Reynolds, & Kush, 2015; McPhee, Claridge, Noorduyn, & Gorter, 2018), mobility (Benner, Hilberink, Veenis, van der Slot, & Roebroeck, 2017; Morgan & McGinley, 2014), and musculoskeletal impairments (Morgan & McGinley, 2014). Recent cross‐sectional studies have identified high rates of pain and fatigue among adults with CP (McDowell, Duffy, & Lundy, 2017; McPhee, Brunton, Timmons, Bentley, & Gorter, 2017). Furthermore, we are starting to recognize mental health outcomes in people with CP, including depression (Gorter et al., 2014) and the associations between physical and mental health symptoms (Benner et al., 2017). A systematic review by Lindsay (2016) described the qualitative literature available on the health implications of young adults (under age 25) with CP (Lindsay, 2016). It identified a need for further research to understand the health outcomes of young people with CP in terms of employment, (intimate) relationships, and identity formation (Lindsay, 2016). The qualitative literature available is primarily on young adults less than 25 years old, so there is also a need for further research on young adults through age 30. Though medical interventions have addressed some of the physical impairments, understanding the social and emotional outcomes for people with CP, as well as the barriers to and facilitators of these outcomes, will enable healthcare practitioners to better care for people CP as they age.

The World Health Organization's International Classification of Functioning, Disability, and Health (ICF) conceptualizes the biopsychosocial factors contributing to health and functioning (World Health Organization, 2002). Our research programme, called *MyStory*, has applied ICF concepts to a lifecourse health development approach (Palisano et al., 2016) to understand the implications of having CP on participation, brain function, and development in adolescents and young adults (aged 16–30 years). Young people with CP felt that the quantitative *MyStory* study did not adequately capture the reality of their lived experiences. Thus, a new integrated research team was formed, applying principles of participatory action research and engaging researchers from the *MyStory* project, clinicians and adults with CP, and other impairments to codesign this substudy: the *MyVoice* study. By enhancing our understanding of the impact of impaired health (particularly the role of chronic stress) on brain function, we hoped to discover interventions to enable youth with CP to achieve full participation in their desired roles throughout life. The aim of *MyVoice* was thus to blend lived experience with rigorous research methods to capture the intricate reality of young adults with CP—their physical, mental, and emotional health—as they navigate their life with a complex health condition.

## 2 METHODS

### 2.1 Population

This study was a qualitative exploration within a large, prospective cohort study exploring “brain‐behaviour correlates of health and well‐being in adolescents and young adults (16–30 years of age) with CP”. The *MyStory* study focuses on physical health, mental health, stress, and overall well‐being in young adults with CP through surveys and quantitative measurements (CanChild Centre for Childhood Disability Research, n.d.). A purposive sample of 16 young people with CP, across all Gross Motor Function Classification System (GMFCS) levels, was recruited to participate in the *MyVoice* study, aimed at understanding the impacts of CP on their lives as they age. The Hamilton Integrated Research Ethics Board approved the study; participants provided informed consent for the MyVoice substudy.

### 2.2 Design and methodology

Interpretive description (ID) is a methodologically eclectic approach to qualitative research for allied health disciplines. It extends beyond simple descriptions of experiences to facilitate the interpretation of complex clinical experiences to inform practice (Thorne, Kirkham, & O'Flynn‐Magee, 2004). ID allows lived experience to be captured as high‐quality research evidence, enabling clinicians to better understand the reality of participants' lives, and was selected as the guiding framework for this study.

### 2.3 Sample

Purposive sampling was used to identify a diverse sample of participants who would be able to provide rich descriptions of the topic of interest (Creswel, 2016). The research team selectively invited participants with diverse gross motor function (determined by self‐reported GMFCS level; Palisano et al., 2008), as well as gender. The only exclusion criteria for this qualitative study were non‐English speakers, as the focus groups (FGs) were conducted in English, and the inability to participate in an FG (physically or over the phone) and to answer open‐ended questions, as this was considered a serious impediment to participation.

### 2.4 Data collection

Two researchers (O. H., research coordinator with a child with Down syndrome, and M. F., PhD candidate with CP) facilitated the FGs. FGs were used to collect data because their inherent group dynamics are an effective way to generate social knowledge related to beliefs and attitudes that underlie behaviour (Thorne, 2008). As many of the topics discussed in the projects can be socially stigmatizing (e.g., mental health, pain management, and stress), especially as they are not often discussed in the context of CP, FGs provide a safe environment for discussion of these topics (Håkanson, Sahlberg‐Blom, Ternestedt, & Nyhlin, 2012; Thorne, 2016).

A semistructured interview guide (see Appendix A) was codeveloped with the research team, with validity of the interview guide established using various perspectives, including those from clinicians and people with lived experience (two adults with CP and a parent of a child with Down syndrome). Discussions were audio‐recorded and transcribed verbatim. The FGs took place in community settings with the option for teleconferencing to accommodate participants with mobility difficulties. Participants with speaking difficulties could complete written answers to questions in the interview guide in addition to their FG participation. Three 2‐hour FGs were conducted with four to seven participants in each. Together, the transcribed information and the facilitators' field notes formed the raw data for analysis. Member checking with participants (via emailed transcript review) was completed to verify accuracy and ensure that responses were faithfully recorded; this was especially important for participants who had speech difficulties. To preserve confidentiality, all identity‐linked information was anonymized; each participant chose a pseudonym to personalize their story comfortably in this paper.

### 2.5 Analysis

One researcher (J. H., a research student with a childhood onset physical disability) read the transcripts and listened to the audio‐recordings several times to ensure the accuracy of the transcription and aid in the analysis. To derive preliminary themes and categories inductively for coding, two researchers (O. H. and J. H.) independently reviewed and coded one FG transcript selected by the research team as representative of the sample. Codes were discussed with the larger research team, following agreement on preliminary codes. J. H. and O. H. coded the remaining transcripts. After initial coding, transcripts were reread to ensure that coding definitions were consistently applied (Corbin & Strauss, 1990; Hewitt‐Taylor, 2001). After all transcripts had been coded, preliminary themes identified, and analytical structure determined, we used peer debriefing and triangulation of research team members that included individuals with CP to ensure credibility.

## 3 RESULTS

Sixteen individuals (across all GMFCS levels) consented to participate in FGs. Gender distribution included seven females and nine males. Age ranged from 17 to 29 years (Mean = 26; Standard Deviation = 3). Participant characteristics are presented in Table 1.

**Table 1:** Participants' characteristics

Pseudonym	Gender	Age	GMFCS Level	Education	Current employment
Joy	F	26	1	Withdrew from college	Unemployed—caring for sick parent
Kathy	F	24	4	Undergraduate student (Social Work)	Student, previously employed at university accessibility office
Mary	F	27	3	Undergraduate (Sociology and Criminology)	Social media writer
George	M	29	1	High school	Unemployed—attending day programme with hopes of working
Muaaz	M	27	5	Undergraduate (Law and Philosophy)	Student
Nicola	M	24	1	College	Works at makeup store (part time job)
Pat	M	29	2	Not discussed	Overnight security, costume designer
Peter (with parent proxy)	M	29	2	High school	Salvation Army store worker (part time)
Josh	M	27		Not discussed	Not discussed
Noah	M	24	2	Undergraduate student (Social Development)	Student, summer camp counsellor
Tristan	M	27	3	Plans on undergraduate (public administration)	Not discussed
Louise	F	28	4	Undergraduate (English and philosophy), Master of Education	Social media blogger and part‐time tutor
Jim	M	29	Not discussed	High school	Summer camp counsellor
Rita	F	17	1	High school student	Student
Mariah	F	27	4	Masters	Minister (contract position)
Nina	F	25	3	Undergraduate	Student

Four main themes emerged from the FGs: (1) health, (2) meaningful participation, (3) impact of the environment, and (4) identity for young people with CP. Subthemes were also identified and included to enhance readability. Relevant quotes for each theme and subtheme are available in Table 2.

**Table 2:** Participants' quotes

Theme	Subtheme	Relevant quotes
Health	Physical health and illness experience	Pat (age 29; GMFCS level II) “I was getting kidney stones on the regular basis and I was getting frequent pain and I was going through countless doctors about the pain … it was giving me a lot of anxiety” Kathy (age 24; GMFCS IV): “I just, for me I do not know if anybody accessed services through [the regional children's treatment centre] when they were younger but I it was everything there—PT, OT, social work and now [in adulthood] everything is like gone.”
	Mental health	Josh (age 27): “I do not really drink but I started drinking because it is the cheapest thing I can afford and it's the easiest way I can escape.”
Meaningful participation	Work and recreation	Louise (age 28; GMFCS IV) “I think that it's really important to me the education that I've been able to acquire and the career aspirations that I have … I work part‐time doing social media managing and blogging for the disability network, so that's about 10 hours a week. I have an undergrad in English and philosophy [from one university]. I also got my master's in education [from another university in a different city] but I have been unable to find work in education. So in addition to my social media job I also tutor whenever I can.”
	Goal setting	
Impact of the environment	Family	Kathy (age 24; GMFCS IV)“My family was really supportive of disability up until I was 18 and I still live at home with them but they do not understand the mental health challenges that I have surrounding disability now because they still see me as that 10 year old … how [do you] you deal with parents that are maybe not where you are at emotionally and how you explain to them the service gaps because my parents are still in like fantasy land and it drives me crazy?” Joy (age 26; GMFCS I): “[I'm] struggling now with my family I've been pushing myself to try and take care of everything for my mom and my sister and my dog and I just find that my body gives out and I get very tired. But my family treats me like I'm perfectly capable of doing anything and that there is nothing wrong with me … but I need a break … My struggle right now is trying to care for my family and trying to care for myself, you know like finding that balance.”
	Intimate and peer relationships	Nina (age 25; GMFCS III): “you should've seen how many people stared at us, like I have a disability and I'm holding the hand of an able‐bodied [guy] like they do not expect that a girl with a wheelchair or walker would be dating … you would think it would be normal because we are just a girl and a guy holding hands but I was in my power chair and everybody looked and I do not understand why it's different” Muaaz (age 27; GMFCS V): “I think other students just assume … that “disability people” wanted to stick together and not expand their social circle. So that's a big problem.” Louise (age 28; GMFCS IV): “99% of my friends are able‐bodied. I like it that way because being surrounded by people who are able‐bodied opens doors for me in what I am able to do.” Nina's (age 25; GMFCS III): “[Although] with my disabled friends we have a lot of shared experiences, I do not really like to have too many disabled friends because it reminds me that I am disabled … Not only do I feel more normal/accepted [when I am with able‐bodied friends] but it teaches the people I hang out with that I am the same as them. Just because I cannot walk the same does not mean that I'm different.”
	Accessibility	Louise (age 28; GMFCS IV): “I had to add a line [to my cover letter] about being in a wheelchair which I really did not want to a do but I got to a point where I got a call‐back from a principal … when we started to talk about the physical accessibility of the environment and I immediately went ‘hmm I cannot’ and he said ‘why not’ and I said ‘because I use a wheelchair’ and he said that ‘I realized you had CP but I did not realize you were in a wheelchair and this is what really got because If I'd known you were in a wheelchair I never would've interviewed you’ … that's kind of a knife in the gut.” Muaaz (age 27; GMFCS V): “A lot of the struggles [around socializing with peers] were trying to get accepted by other people and to find activities that do not involve going to places that are inaccessible or making places more accessible so that people in wheelchairs … the provinces need to work together to make laws and make sure that places more accessible.”
CP and identity		Rita (age 17; GMFCS I): “It's something that's hard for a lot of people with disabilities, so I take pride in the fact that I play a lot of sports.” Josh (age 27): “My CP is somewhat invisible. And that's been a big hassle in some ways because I have some people who understand my CP but want to treat it like it's not there. Even my immediate family … They want to play the disability card when it suits me not when it does not suit me. And that does not really gel.” Mary (age 27; GMFCS III) “It means we can have different identities as people so like in saying that I have CP and I'm a minister and I'm gay. Any of those identities clash with each other or cause stress. We have different identities.”

### 3.1 Theme 1: Health

Body structure and function aspects of health were discussed extensively in all FGs. For many participants, this was their first time discussing their physical and mental health challenges with peers, and several participants were surprised that other young adults with CP faced similar challenges.

#### 3.1.1 Subtheme (i): Physical health and illness experience

Fatigue, pain, and anxiety (often about pain) were reported in participants' daily lives, activities, and functioning. Several participants described painful and often debilitating spasms that interfere with sleep, work, and daily functioning. Other pain reports included back pain due to sitting or physically demanding jobs. There were more acute causes, such as pain from surgery and recurring kidney stones that went undiagnosed.

Getting care for pain and physical health issues was shaped by the experience of having CP. Many participants described challenges accessing adult healthcare providers (HCPs), especially HCPs who understood their CP in the context of their physical and mental health. There was also extensive discussion about the lack of adult services.

#### 3.1.2 Subtheme (ii): Mental health

Participants also discussed the impacts of mental health on their daily lives. Stressors both directly related to CP (e.g., making healthcare decisions and surgeries) and unrelated (school, work, relationships, finances, etc.) impacted the mental health and general well‐being of almost all participants. Depression and anxiety were frequently reported among participants, though many lacked a formal diagnosis and consequently lacked support. One poignant example is Joy's experience: She spoke extensively about her inability to access services and how this influenced her mental health and well‐being. She experienced fatigue and mental health ramifications associated with CP but was unable to access services and funding because she had “mild” CP and lacked a formal mental health diagnosis. Joy also shared her experience of homelessness. Participants were open and honest in sharing variable coping strategies to manage their physical and mental health challenges, including two participants reporting excessive substance use. Others reported using pain killers, medicinal cannabis, acupuncture, and massages to cope. A few participants described other coping strategies including reading books, being physically active, art, video gaming, or voice acting. Social connectedness was emphasized as an important coping strategy.

### 3.2 Theme 2: Meaningful participation

Most participants chose to introduce themselves with the different activities in which they participate—including work, school, and recreational opportunities (as opposed to introductions based on diagnosis). It became obvious that participation was just as important, if not more so, as their underlying health condition. These various activities were imperative for the young adults to achieve “normality.” Participants did not talk about goals related to their CP (walking, achieving better gross or fine motor function, etc.); rather, they discussed goals related to sport, education, employment, and other recreation.

#### 3.2.1 Subtheme (i): Work and recreation

As examples of participation, sporting activities ranged from soccer, ice hockey, and field hockey to various parasports and adaptive fitness programmes. Artistic activities were also identified by a range of participants; for some, it was a method of coping with the lack of relationships or pain. One participant described his artistic endeavours as “the most positive thing” in his life. For one costume designer, the artistic hobby provided an unfolding employment opportunity.

In most instances, participants were self‐employed or had entry‐level jobs such as customer service, social media, or camp counselling. Many chose to include in their introductions the positions they have been searching for but have yet to get. Participants emphasized the importance of participation to their well‐being and as an important aspect of their identity.

#### 3.2.2 Subtheme (ii): Goal setting

Goal setting was a notable subtheme of participation. Personal goals focused on educational and occupational issues, as well as those related to having a family and living independently. Because several were unemployed, underemployed, or volunteering, they were seeking more stable, higher level, or less physically demanding positions. Interestingly, some participants also shared a variety of social goals relevant to their collective identities. Those included increasing social support services for adults with CP, LGBTQ+ advocacy, making a more compassionate and understanding society, and improving accessibility in the community. Despite articulating a variety of personal and social life goals, only a few participants tied their goals explicitly to their CP.

### 3.3 Theme 3: Impact of the environment

#### 3.3.1 Subtheme (i): Family

Starting from early childhood family was a significant influence and an environmental factor in participants' lives. For some, family was an enabler of independence and goal accomplishment. Others were cognizant of the continual dependence on the family. Some participants reported disagreements with their parents, including frustration about parents not understanding emotional and mental health needs of their adult children.

Participants reported complex dynamics with (in) the family that were influencing their experience and participation as adults with CP. Joy discussed her role as her mother's primary caregiver, a role further complicated by her CP. The discussions with participants emphasized the importance of family in the lives of adults with CP; family served as both a barrier and facilitator to their well‐being.

#### 3.3.2 Subtheme (ii): Intimate and peer relationships

Several participants alluded to an interest in having intimate relationships. Participants described previous relationships, many of which were with able‐bodied individuals. Some participants were currently in a relationship, and many discussed the challenges of asking for help from their significant other. Two participants who self‐identified as LGBTQ+ describe how intersections influenced them (see further CP and identity). Several participants shared their thoughts and experience with stigma and relationships.

Relationships with peers were very important for participants' well‐being and positive identity. Some members of the group reported very few problems making friends with peers and alluded to the positive influence of the social relationships on their well‐being. On the other hand, some participants reported challenges making friends, especially at university. Most participants described having a large proportion of “able‐bodied” friends and recognized the importance of having a balance between disabled and able‐bodied friends. Some participants also described becoming less anxious about being judged by people as they aged, making it easier to make friends as adults.

#### 3.3.3 Subtheme (iii): Accessibility

Accessibility in public, social, and recreational places where people with CP could intermingle with others was an extensively discussed environmental factor. Accessibility was particularly pronounced when participants tried to secure sustainable employment and financial independence. Some of the reported challenges are similar to those experienced by their peers without CP (e.g., finding full‐time teaching positions and balancing social life with long hours). Many limitations, however, were due explicitly to the CP: pain and having to rely on heavy pain killers, other codisabilities (visuospatial and/or learning), and lack of meaningful inclusion in diversity enhancement programmes. A distinct example of discrimination in the hiring process came from Louise who was applying for teaching jobs and was rejected because of her wheelchair use.

Although several participants discussed their ability to participate in social and recreation opportunities, many depicted a lack of physical accessibility as a significant barrier to participation. This was especially true among wheelchair users. Many participants discussed at length the need for increased physical accessibility, especially in social environments and meeting places (such as bars and restaurants). Accessibility was discussed at length in all FGs.

### 3.4 Theme 4: CP and identity

As participants had a wide range of GMFCS levels and personal experiences with CP, their apparent desire to identify as part of a group of individuals with CP varied a fair amount. Only two participants immediately identified their CP as a part of their introductions: Kathy and Peter (whose father spoke on his behalf). Other participants had hints or subtle undertones of the effects of CP on their self‐concepts; however, only upon further probing (e.g., Why did you define yourself in this manner?) did participants explain the effects CP has had on their identifiers. For example, one person introduced himself as an artist and later revealed that “hospital art” was important to him as it helped him get through his surgeries and frequent childhood hospitalizations. On the other hand, Rita, a high school student with very mild CP, chose to introduce herself as a soccer and field hockey player because she felt proud that she could play sports while realizing that it is hard for others with disabilities.

Several participants reported challenges when navigating their experience related to their CP/disability. The challenges were particularly pronounced for those participants with the mild and so‐called “invisible” form of CP.

Intersections of identity were particularly pronounced in the discussions in one FG. A very telling example came from one participant who identified as LGBTQ+ and was quick to educate another participant about the meaning of identity intersections.

## 4 DISCUSSION

This qualitative study, initiated in response to participants' desire to share their lived experiences, illustrates these experiences and some consequences of growing up with CP—namely, the physical and mental health challenges, the importance of meaningful participation to improve quality of life, the impact of the environment on well‐being and health, and identity formation. Across GMFCS levels, participants shared challenges related to accessibility of healthcare, social/environmental supports, relationships, and sustainable employment.

As children with CP become adults, their health needs extend beyond physical and mobility impairments: Pain, fatigue, and mental health challenges become more prominent (Lindsay, 2016; Mesterman et al., 2010; Sienko, 2018). The participants described how these challenges, along with other comorbidities, interfered with functioning including sleep, school, work, social, and home life. Some sought medical attention; however, many lost hope, going without a diagnosis, treatment, or both, sometimes because HCPs did not believe their experiences or the level of pain experienced. This lack of adult HCPs who understand the broad implications of having CP caused some participants additional stress and anxiety. The FG discussions identified a gap between participants' health needs and their care in the adult healthcare system. HCPs trained in managing adults with CP are needed to improve the care and quality of life of people with CP. Additionally, there needs to be more awareness that anxiety and depression, pain, fatigue, and weakness are common in people with CP—but not automatically part of CP—and that environmental and attitudinal factors may play a pivotal role in the development of secondary health issues and accelerated ageing with disability (Putnam, 2002; van der Slot et al., 2010). Hence, there is a need for a preventative approach focusing on healthy development and early interventions to optimize function and health (Bogart, 2014). Body structure and function challenges threaten youths' ability to participate in activities of daily living, have a career, and engage socially, thus threatening their ability to form positive identities and live meaningful lives as adults.

Work participation was a major subject of the FGs, with emphasis placed on barriers to engaging in meaningful, education‐appropriate employment. This is in line with recent literature assessing employment among persons with average intelligence with CP that found higher rates of unemployment, especially for those requiring workplace accommodation (Verhoef et al., 2014). There is a paucity of research about how youth with CP navigate the process of finding meaningful, education/skill‐appropriate employment. Their descriptions and realities of finding and keeping jobs outline challenges and strategies used to mitigate barriers related to disclosure, requesting accommodation and pursuing further education. It is imperative for HCPs to be cognizant that, like everyone else, individuals with CP desire to work. A recent scoping review covering multiple disabilities underscores the importance of paid work, as it serves as a source not only of financial independence but also of socialization and positive identity (Saunders & Nedelec, 2014). The challenges participants described when trying to secure a financially sustainable position reflect the trend of reliance on disability benefits and social assistance (Verhoof, Maurice‐Stam, Heymans, & Grootenhuis, 2012). Life skills programmes and resources are needed to help young people acquire interviewing skills and strategies to manage disability disclosure and navigate job applications. Facilitating employment opportunities and skill development for young people with CP not only benefits the well‐being of the person and their family but also enables them to rely on their own income rather than social services income supports.

Environmental conditions, including relationships, supportive people, and environmental accessibility shaped participants' health, well‐being, and identity. Previous literature suggests that youth with disabilities are at a higher risk of being bullied and socially excluded and often lack friendships (Lindsay & McPherson, 2012). Friendships are a key focus of positive relationships and social and emotional development (Rosenbaum & Gorter, 2012). The participants articulated the need for, and importance of, friendships with peers both with and without disabilities. They also highlighted the role of having accessible environments for learning, working, and socializing. Additionally, during FG discussions, participants offered each other their own strategies and personal support, including connecting with each other beyond the FGs. In and of itself, this is important to note, as it may indicate a lack of opportunities to discuss potentially stigmatizing issues with peers. Service providers need to include discussions about friendships and relationships in their care of children and young adults with CP, understanding that there are barriers that prevent typical socialization in this group. Paediatric and adult HCPs are encouraged to support mainstream socialization opportunities; at the same time, supports are necessary to promote interactions with peers who have similar health conditions. Such efforts should also include discussions on potential or actual challenges the young adult may experience related to relationships and use a strengths‐based approach (i.e., sharing practical strategies related to social interactions). It is therefore critical for HCPs to address and advocate for these issues, because social isolation and exclusion are associated with higher rates of anxiety, depression, and poor quality of life (Seng, Lopez, Sperlich, Hamama, & Reed Meldrum, 2012).

## 5 LIMITATIONS

This qualitative study involved 16 participants at one point in time. Although participants have a wide range of GMFCS levels, the findings must be interpreted with caution as individuals with CP do not automatically share the same life experience. ID, a relatively new qualitative study design, was used to inform the study. There are limitations to the use of ID and qualitative research, but it allowed for interpretation of participants' lived experiences through various lenses—including those of clinicians, people with CP, and researchers with CP. The semistructured interview guide was created with people with CP, using a patient‐oriented research approach, to ask questions related to patient‐important outcomes. So, although not formally validated, this interview guide has face validity for our population and the research question being explored. Another limitation of the project is the utilization of FGs instead of individual interviews. However, the FGs helped facilitate discussion about stigmatizing topics and provided a benefit to members participating in the study.

## 6 CONCLUSION AND FUTURE DIRECTIONS

Child health and rehabilitation providers strive to promote optimal health and full participation of individuals with CP. To do this, we must go beyond understanding CP as a medical condition with its associated impairments and move towards a better understanding of the transactional processes between people growing up with a condition like CP, personal and environmental factors, and health outcomes. This study further elucidates the need for improved care for young adults with CP, who may require the support of multidisciplinary adult health teams and community services. Further research should strive to understand the links among identity, attitudes, mental health, and support seeking in adults with CP and best ways to support individuals with child‐onset conditions as they navigate their “health identity” development (Eccleston, Williams, & Rogers, 1997; Fox & Ward, 2008; Morley, 2008; Seng et al., 2012). As one output of this study, knowledge translation activities have already been started to increase public awareness, including a webinar initiated and cocreated by young people CP about the importance of mental health. It is hoped that this study will contribute to our understanding about the lifelong health implications of having CP and that findings like these will inform policy and practice with the goal to improve programmes and services to better facilitate healthy development as an adult.

## APPENDIX A SEMISTRUCTURED INTERVIEW GUIDE

1. **Introductions and one's sense of him/herself**—Let us begin with brief introductions.
  - As part of your introductions, please name two to three things that are important in your life, things that you do on a regular basis, and that perhaps define who you are.
  - Let us talk a bit more about why you chose to introduce yourself as … This question takes us to the concept of identity, your sense of who you are. All of us wear many different hats in our life and play many different roles. In other words, why do your descriptions of yourself matter to you?
    Probe: In some cases, some of these different roles (defined by one's age, gender, ability, education level, religion/spirituality, race, ethnicity, sexuality …) intersect or overlap in one person.
  - Do any of those descriptions (or intersections) perhaps represent areas of struggle (or cause you stress or anxiety)? Why?
    Probe: If needed with examples: (non)‐White (non)‐Christian male or female with CP; a person with CP interested in relationships with the same sex, opposite sex, or both.
  - How could any of those struggles be addressed? Or perhaps someone would like to share an example of how you are managing such a struggle without getting overly stressed out or anxious?
2. **Making sense** of the *MyStory* study (quantitative part) and beyond
  - Let us talk about **the study** that you have been participating in and **what it means to you.** We have asked you many questions related to anxiety, depression, pain and fatigue, quality of life, and family functioning,
    - Does any question or area stand out as most useful (or least useful) for you personally?
    - Would you be interested in knowing your scores—your group scores or individual scores?
    - Which of those areas (anxiety, depression, pain and fatigue, quality of life, and family functioning) would you like to know the results for?
    - Are there any areas or questions you feel the research team should have asked you about or you would like to learn more about?
  - Let us talk about stress and pain a bit more
    - Are there any life domains that we have not discussed that concern you or cause you most stress or anxiety on a regular basis? For example, healthcare, living, financial arrangements, mobility, educational, occupational, and recreational options. Probe: How much choice or option you have in any of those domains? How do you feel about the degree of choice (or lack thereof)?
    - Would you like to share your coping strategies with stress or anxiety?
    - How can stress and anxiety be minimized in your opinion?
    - Do you feel you have access to mental health services if you need it? Any concerns or barriers that you would like to discuss?
    - How about pain and/or fatigue? Who would like to discuss the role they play in your life? How do you manage pain or fatigue? Do you feel you can discuss it with your doctor and get help? Any concerns? Probe: Alternative therapies (e.g., massage, hydrotherapy, hypnotherapy, cognitive therapy, music, or arts therapy)? What place do they have in your life? Any concerns about or barriers to their use?
    - Can you think of any emotional aspects that can make pain worse or less?
  - **Relationships** play a big role in our lives as they include relationships with your family, friends, or colleagues; healthcare providers; and also relationships with (intimate) partners. Sometimes, relationships make you happy and can help you cope with stress, pain, and fatigue; sometimes, they actually cause stress and anxiety. Can you describe any of those scenarios?
3. **Concluding/moving forward**—Let us conclude this great discussion with these points:
  - What recommendations do you have for us to take back to our research?
  - What role would you like to play in the research process? For example, would you be willing to form an advisory group and/or networking group that could help researchers and policymakers and other young adults with CP? What format would work best for you—online (e.g., Facebook) or any other?
